# The selective flow of volatile organic compounds in conductive polymer-coated microchannels

**DOI:** 10.1038/srep42299

**Published:** 2017-02-13

**Authors:** Faramarz Hossein-Babaei, Ali Hooshyar Zare

**Affiliations:** 1 Electronic Materials Laboratory, Industrial Control Center of Excellence, Electrical Engineering Department, K. N. Toosi University of Technology, Tehran 16315-1355, Iran

## Abstract

Many gaseous markers of critical biological, physicochemical, or industrial occurrences are masked by the cross-sensitivity of the sensors to the other active components present at higher concentrations. Here, we report the strongly selective diffusion and drift of contaminant molecules in air-filled conductive polymer-coated microfluidic channels for the first time. Monitoring the passage of different target molecules through microchannels coated with Poly(3,4-ethylenedioxythiophene):poly(styrenesulfonate) (PEDOT:PSS) revealed that contaminants such as hexane, benzene, and CO pass through the channel unaffected by the coating while methanol, ethanol, and partly acetone are blocked. The observations are explained with reference to the selective interactions between the conductive polymer surface and target gas molecules amplified by the large wall/volume ratio in microchannels. The accumulated quantitative data point at the hydrogen bonding as the mechanism of wall adsorption; dipole-dipole interactions are relatively insignificant. The presented model facilitates a better understanding of how the conductive polymer-based chemical sensors operate.

Monitoring the concentrations of hazardous gases in air and ensuring that they do not exceed safe levels is a mandatory practice in many industrial environments^1^,^2^,^3^,^4^. Different methods have been proposed for the selective detection and identification of gaseous analytes with simple instruments^5^,^6^,^7^,^8^,^9^,^10^,^11^,^12^,^13^,^14^, but the task is hard to fulfill as different contaminants often have vastly different safe levels^15^,^16^,^17^,^18^. For instance, the safe level for benzene, a carcinogenic volatile organic compound (VOC) and carbon monoxide (nicknamed “the silent killer” where the authors reside) is lower than 10 ppm while the allowable concentration of methanol, ethanol, and acetone is 10^2^ times higher^19^,^20^,^21^. The sensitivity of commonly used tin oxide-based gas sensors to these contaminants is of the same order of magnitude (these sensors are typically more sensitive to ethanol than benzene or CO)^22^,^23^,^24^, and, hence, the presence of VOCs such as ethanol at a safe level will totally block the detection of benzene or CO at very dangerous levels. Gas chromatograph coupled to a mass spectrometer is the system utilized for these analyses^25^,^26^, but these devices are massive, expensive, incapable of online operation, and dependent on expert attendance.

Biomarker VOCs such as acetone, ethanol, isopropanol, 2-butanone, benzene, toluene, hexane, and isoprene, are related to specific biological occurrences^27^,^28^,^29^,^30^,^31^,^32^. The detection of benzene and hexane in 1–160 ppb levels in exhale gas, for instance, has been connected to lung cancer^33^,^34^,^35^,^36^,^37^,^38^. On the other hand, both ethanol and acetone are naturally present in exhale gas at the 100–900 ppb concentration range^38^,^39^,^40^,^41^, which can block detecting the targeted markers with appropriate biosensors. Fast screening of such target contaminations requires highly selective sensors and/or active filtering of the contaminated air prior to sensor exposure^42^,^43^,^44^,^45^,^46^,^47^. Solving these technical problems and many other similar difficulties requires the gas sensor to be coupled to strongly selective filters^48^,^49^,^50^. For instance, the bottleneck in solving the problem of detecting masked CO would be the incorporation of a filter which attenuates all the commonly present VOCs and allows the sensor be exposed to the carbon monoxide contamination.

The integration of a gas sensor with a microfluidic channel has been shown to result in a device for gas microanalysis^5^,^6^,^51^. In such a device, target gases travel through the length of the microchannel prior to affecting the sensor. Different target gases result in different temporal response profiles which are utilized for their recognition. Here, we report the fabrication of a microchannel with conductive polymer coated walls, and investigate the flow of air carrying different contaminants through the fabricated channel by integrating it with a general gas sensor. The results obtained for a microchannel with Poly(3, 4-ethylenedioxythiophene)-poly(styrenesulfonate) (PEDOT:PSS)-coated walls show that the channel allows unobstructed passage of carbon monoxide, benzene, hexane and hydrogen while strongly blocking VOCs such as methanol, ethanol, and acetone. The presented model describes the selective adsorption of the airborne molecules to the PEDOT:PSS surface.

## Results and Discussion

**Figure 1:**
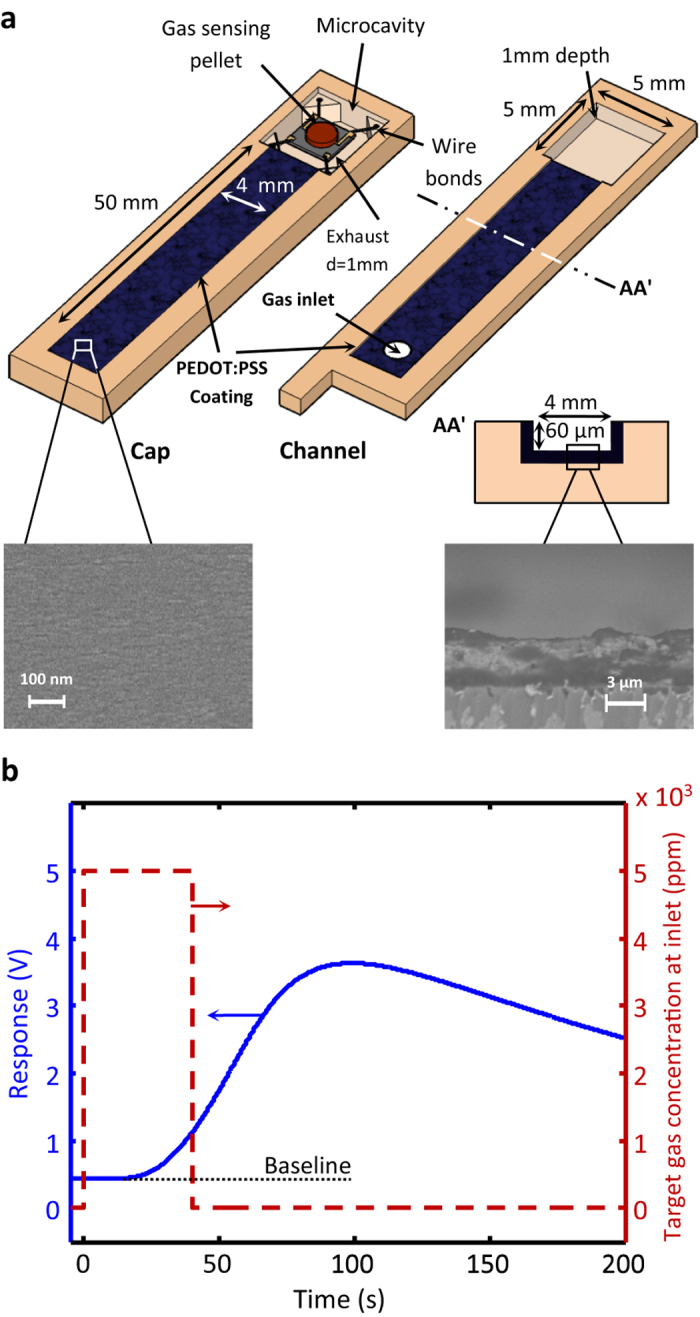
The device structure and the test procedure utilized. (**a**) The schematic presentation of the PEDOT:PSS-coated microfluidic channel integrated with a gas sensor, and (**b**) a typical temporal response recorded upon exposing the inlet of the channel to the contaminated air from 0–40 s; during the rest of the 205 s recording time, the inlet is connected to the clean background atmosphere.

The investigated devices are fabricated by integrating a microchannel with a generic gas sensor on poly(methyl methacrylate) (PMMA) and borosilicate glass substrates^5^,^6^. The sample structure, schematically presented in Fig. 1a, comprises a PMMA channel with walls coated with a micron-thick layer of PEDOT:PSS; and the control sample is a similar device with uncoated channel walls. (How we arrived at this concept and selected the coating material is briefly described in the Supplementary Section 1). At each experiment, the diffusion and flow rates of an airborne contaminant (target gas) with a predetermined concentration in two devices different only in the channel wall coating are compared at similar conditions. The fabrication and test procedures are described in Methods below. The protocol utilized for recording the target gas diffusion/flow rates through the sample microchannels is presented in Fig. 1b. The background gas is the ambient air uncontaminated with target gases (clean air) which fills the test chamber and the microchannels prior to the test. Each test cycle starts at *t* = 0, when the inlet of the channel is exposed to the target gas-contaminated atmosphere. At *t* = 40 s, the inlet is reconnected to the clean air. The response of the gas sensor incorporated in the microcavity located at the channel end (Fig. 1a) is continuously recorded from *t* = -5 s to *t* = 200 s. The relationship between such temporal response profiles and the diffusion rates through the channel has been the subject of discussion in a number of publications from our laboratory^52^,^53^,^54^, but in the present work such responses are utilized only for comparing the permeabilities of the test and control microchannels to the target gas.

**Figure 2:**
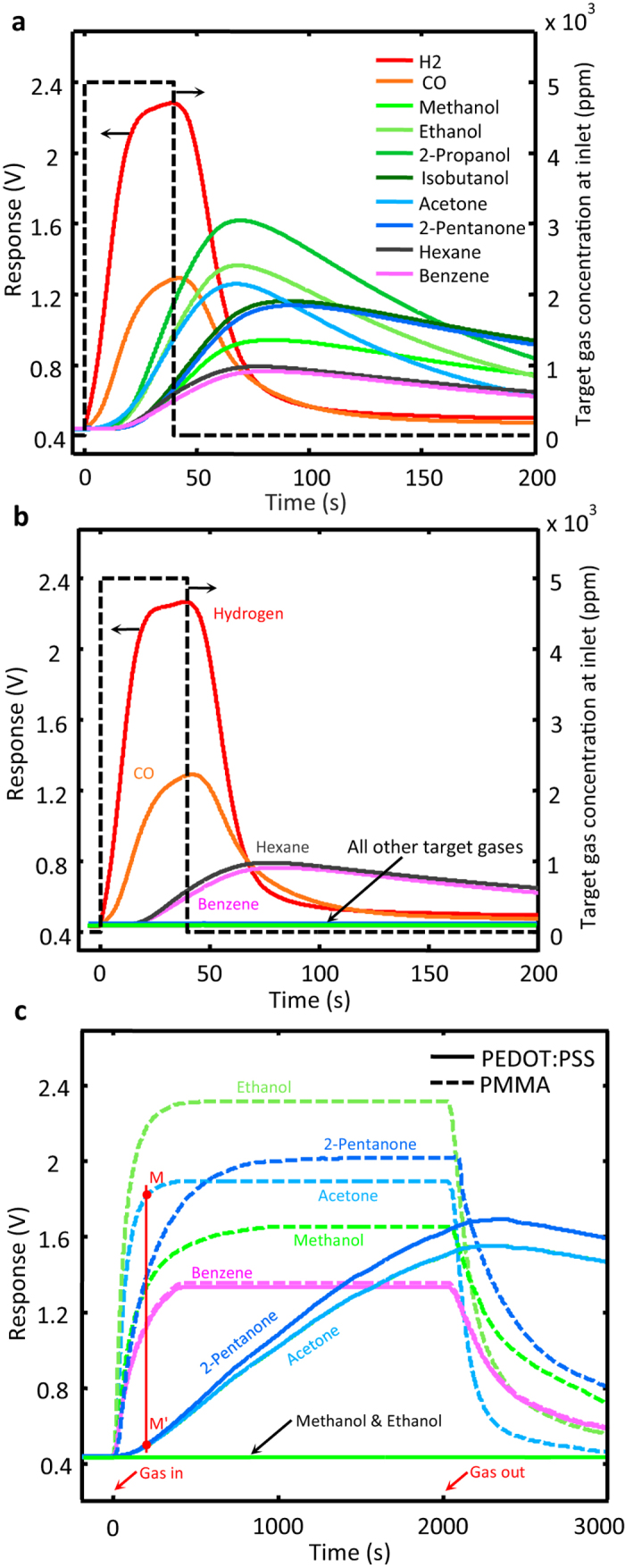
Temporal sensor responses. Recorded for the stated contaminants using the control (**a**) and the coated (**b**) channels with 40 s exposure times; long-term (2000 s) exposure results for both devices are presented in (**c**). The red vertical line in (**c**) is drawn for determining the filtering power of the device with respect to acetone; the result is F_acet._ = 30.

The first set of experiments are carried out in isobar conditions, i.e. the test chamber is at the same pressure as the surrounding atmosphere and the only driving force for the target gas flow through the channels is the concentration gradient. The temporal profiles obtained for the diffusion progress rates of 10 different contaminants in the control channel are given in Fig. 2a. Similar experiments carried out for the PEDOT:PSS-coated channel resulted in the profiles presented in Fig. 2b. According to Fig. 2a and b, the wall coating hardly affects the diffusion rates of H_2_, CO, hexane, and benzene contaminations while drastically reduces the diffusion progress rates of methanol, ethanol, acetone, isopropanol, isobutanol and 2-pentanone. The recorded responses of a single device, at constant temperature (25.0 °C) and controlled humidity (22 ± 1%) are reproducible. The responses recorded in 8 different work sessions for 4 different target gases using a PEDOT:PSS-coated channel and a control channel are presented in Fig. S2a and b, respectively. (see Supplementary, Section 2). From one device to another, however, channel fabrication errors and the differences in the sensing element calibration cause minor temporal shifts and amplitude alterations, but the gas filtering performance of the device remains intact. Aging does not alter the gas filtering strength of the PEDOT:PSS-coated channels either; a coated channel sample, kept in clean dry air at room temperature and tested once a week since 10 months ago, operates like new.

Increasing the exposure times to the target contaminants does not alter the general trend of the results obtained for benzene, ethanol and methanol, but it changes the situation for acetone and 2-pentanone as these gases become detectable at the sensor end for exposure times above 100 s (instead of our standard 40 s). The results of the extended diffusion tests for methanol, ethanol, acetone and 2-pentanone, with exposure times as long as 2,000 s, are presented in Fig. 2c where they are compared with the similar results generated using the uncoated channel. Considerations based on the results presented in Fig. 2c and other similar data translate into a comparative order of magnitude relationship for the filtering power (F) of the PEDOT:PSS-coated channel over the different target gases. This parameter is defined based on the data shown in Fig. 2c as the ratio of the target gas concentrations at the ends of the control and coated channels measured at the time the former attains 95% of its maximum level. In the case of acetone, for instance, F is obtained from dividing the base line-corrected response levels in point M to that in point M’ (see the plots related to acetone in Fig. 2c). F values of the test channel for different target gases are given in Table 1. Experimental limitations do not allow the measurement of F values above 800. For instance, in the response profiles presented in Fig. 2c, the response level for ethanol even 2,000 s after exposure is immeasurably small. For the observation of the wall adsorption saturation and a rough estimation of F_eth_, experiments with 13,000 s exposure times were devised, which resulted in 9,000 for F_eth_. The results are presented in Fig. S3, Section S3 of the Supplementary.

**Figure 3:**
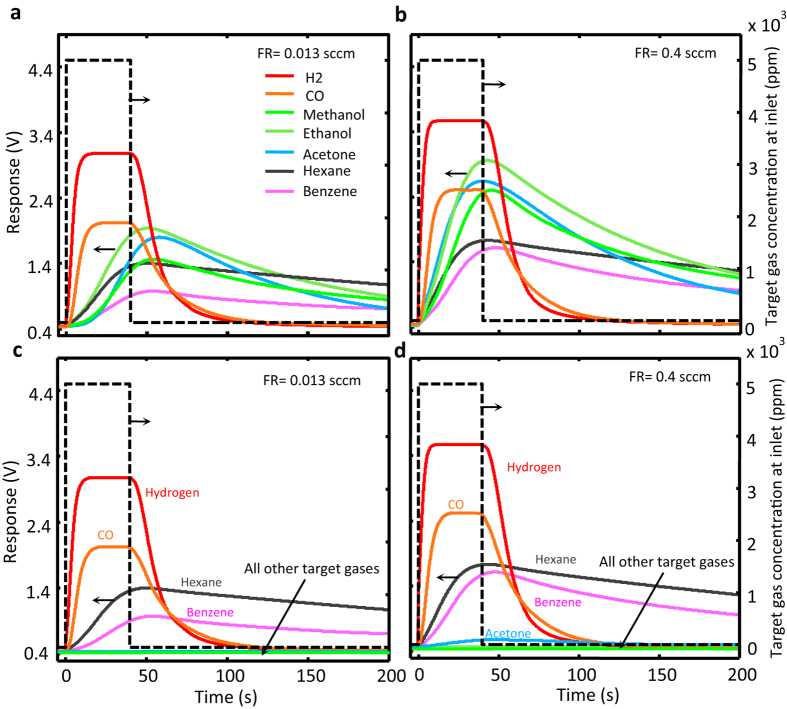
The temporal responses of the sensor to the target gas when a contaminated airflow is established. Along the microchannel for 40 s, using both control channel (**a** and **b**) and coated channel (**c** and **d**) at the stated flow rates. The dotted line presents the imposed temporal target gas variation at the channels’ inlet.

Another set of experiments were carried out with a positively biased chamber pressure with respect to the surrounding atmosphere. The positive pressure of the chamber causes a contaminated air flow along the channel from the inlet to the exhaust. The flow rate in the channel is dependent on and controlled by the magnitude of the established pressure difference which is determined with a U-shaped manometer. The estimated speed of contaminated air within the channel in different experiments is within the 0.1–3 cm/s range (equivalent to 0.013–0.4 sccm at 1 atm and 0 °C). The sensor response recorded for both the control and the coated channels at different flow rates are given in Fig. 3a–d showing the accelerated contaminant passage through the channel. A temporal comparison of the events shown in Figs 2a and 3b clarifies the faster passage of the contaminants in the latter set of experiments; for example, the response to ethanol reaches its maximum in 68 s at zero air flow rate (Fig. 2a), while the same is achieved in 43 s at a flow rate of 0.4 sccm (Fig. 3b). The trend is the same for all the contaminants tested in the control channel. The same is true for H_2_, CO, hexane, and benzene in the coated channel, as well, but none of the other VOCs reach the channel end, even at the 0.013 sccm flow rate within the 200 s recording time, as if the channel were blocked for these VOCs. Ethanol, for instance, is not detected at the channel end even 200 s after establishing the flow.

**Figure 4:**
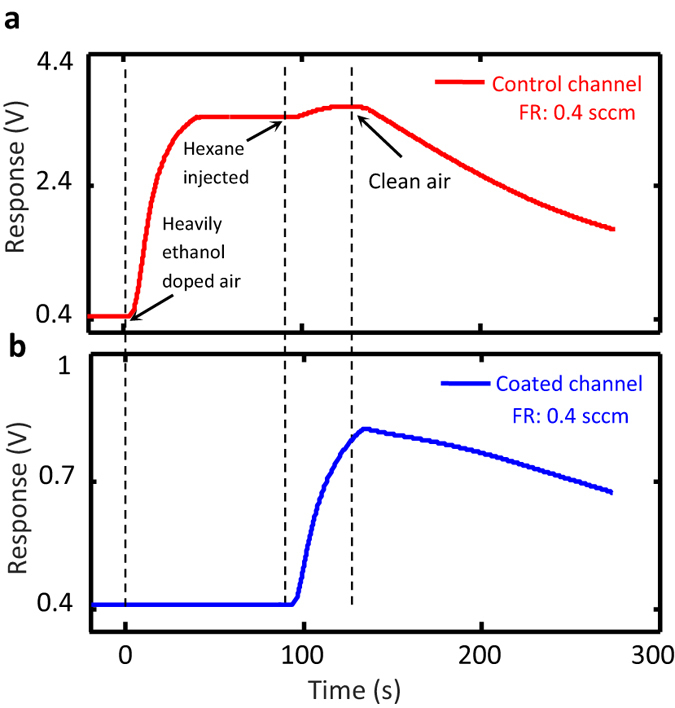
Demonstration of the selective flow of contaminants through the coated microchannel. The temporal responses of the gas sensors located at the ends of the control (**a**) and the coated (**b**) microchannels upon establishing a continuous flow of 10000 ppm ethanol-contaminated air at *t* = *0* through them; in both cases, 1000 ppm of hexane is introduced to the test chamber at *t* = 90 s.

To demonstrate the effectiveness of the filtering action, the ethanol contamination level of the air in the test chamber is increased to 10,000 ppm and a forced flow of 0.4 sccm is established through both control and coated channels. While no sign of ethanol is detected by the sensor at the coated channel end after 90 s, the response at the control channel’s end acquires its maximum level in ~40 s. Then, at *t* = 90 s, 1,000 ppm of hexane is injected to the heavily ethanol contaminated test chamber, which is detected 2 s later by the sensors at both channels’ ends. The results are presented in Fig. 4a and b for the sake of comparison. In the control channel the presence of 10,000 ppm of ethanol upstages the appearance of hexane at 1,000 ppm level (Fig. 4a), while in the case of coated channel the whole response occurs upon hexane injection. Similar results are obtained when hexane is replaced with either carbon monoxide or benzene.

The filtering action is more effective in the channels with smaller cross-section, and the effect is profound in the microfluidic size range. The selective absorption of the VOCs by the PEDOT:PSS-coated channel walls is clearly amplified by the well-known microfluidic features

of the channel; in millimeter-thick channels of the same length, the gas filtering properties of the coating materials are concealed. In other words, in mm-sized channel (e.g. a channel similar to those shown in Fig. 1a, but with a 1 mm cross-sectional height instead of 60 μm) the value of F for all gases examined is 1.0. The observed selectivity levels in the microfluidic channels are surprisingly high. For instance, our estimations based on experimental results obtained for our 50 mm long PEDOT:PSS-coated microchannel revealed that the separation factor between ethanol and hexane, defined as the ratio of F_hex_ to F_eth_, was larger than 10^3^. This places further emphasis on understanding the mechanism of gas adsorption to the surface of PEDOT:PSS layers.

The previous model put forward to describe the effect of VOCs and humidity on the electrical conductivity of PEDOT:PSS^55^,^56^ underline the electric dipole of the target molecule as the key element in the adsorption tendency to PEDOT:PSS surface^57^,^58^. However, some of the above presented experimental results summarized in Table 1, challenge this stance. For example, the molecular dipole moment of acetone is significantly larger than those of ethanol and methanol, while its tendency to get adsorbed by the coated channel walls is at least 30 times weaker than ethanol. Another example is the case of CO, whose passage through the channel is not affected by the PEDOT:PSS coating (compare CO-related response profiles presented in Fig. 2a and b).

**Figure 5:**
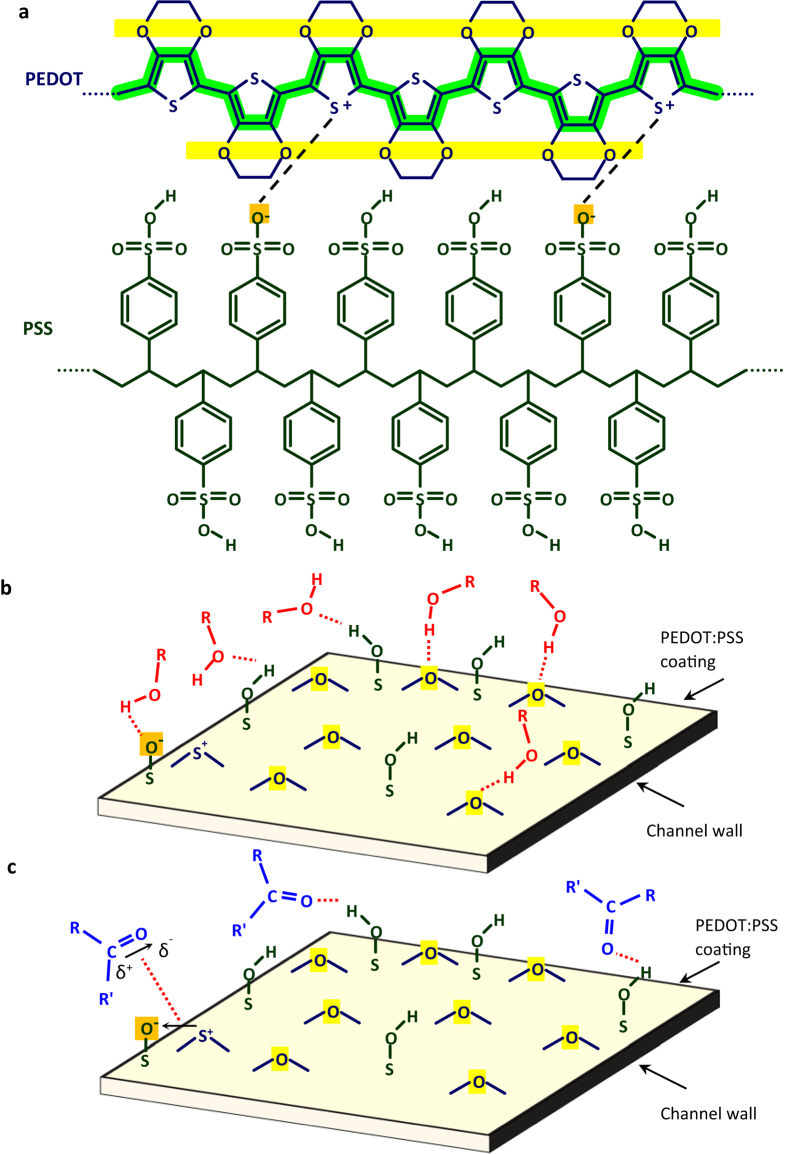
The schematic description of the presented surface adsorption model. (**a**) The macromolecular chains constituting a PEDOT:PSS layer, and the different mechanisms of ethanol (**b**) and acetone (**c**) adsorption to the coated channel walls.

The molecular structures of the two components constituting PEDOT:PSS are given in Fig. 5a. Based on this structure, it is easy to envisage the abundance of the oxygen atoms strongly bonded to the polymer chains at the surface of the PEDOT:PSS layers. The concept is schematically presented in Fig. 5b. These oxygen species, highlighted in Fig. 5a, constitute a considerable portion of the surface structure and are at the most suitable condition for hydrogen bonding with the airborne molecules able to form such bonds. Among such molecules are the alcohols examined in the present work, but their vast spectrum covers molecules ranging from ammonia to proteins and DNA. Considering the colossal surface population of the oxygen atoms covalently bound to the solid polymer (see Fig. 5b), we attribute the observed strong channel wall absorption of these alcohols to their hydrogen bonding with the surface oxygen species. The PEDOT:PSS coating-caused channel adsorption is higher for methanol and ethanol than the propanol and butanol isomers examined. This unexpected result is attributed to the steric hindrance originating from the structural geometry of the molecules; in larger molecules the probability of favorable impact between the hydroxyl group and the wall surface is smaller.

Ketones, regardless of their large molecular dipoles, are unable to form hydrogen bond and their adsorption to PEDOT surface is mainly owing to the dipole-dipole forces, which are of lower bonding energy. On the other hand, ketone molecules can act as the hydrogen bond acceptor, but the surface concentration of the suitable sites for such bonding, sulfonate groups in the unionized form on the PSS macromolecular chain, is considerably less than the above mentioned sites for the hydrogen bonding of the alcohols. The suggested mechanisms of adsorption via both hydrogen bonding and dipole-dipole interactions are schematically illustrated in Fig. 5c. Carbon monoxide, however, is unable to take part in hydrogen bonding and its molecular dipole, 0.12 D, is significantly smaller than that of acetone. These features describe the observed unobstructed flight of the carbon monoxide molecules through the channel. The cases of benzene and hexane are also similar; they are unable to take part in hydrogen bonding and their permanent molecular dipole is zero. Accordingly, the passage of these VOCs through the channel is not affected by the PEDOT:PSS coating of the microchannel.

According to the presented model, methanol and ethanol molecules form hydrogen bonds with the oxygen atoms of the PEDOT macromolecular chain. These atoms, highlighted in Fig. 5a, are closely connected to the charge transport channel along the polymer chain (also highlighted in Fig. 5a). These hydrogen bonds are expected to disturb the charge distribution along the conduction channel and reduce the hole mobility and, hence, the electrical conductivity in the PEDOT:PSS layer. Acetone, on the other hand, accepts hydrogen bonding from the sulfonate group and the charge transportation route along the PEDOT chain remains mainly untouched in its presence. These predictions were verified by the results of our preliminary conduction measurements on PEDOT:PSS layers carried out in the presence of these VOCs. Indeed, the presence of both alcohols cause conduction reduction in PEDOT:PSS layers while the presence of acetone, benzene, and carbon monoxide had no detectable effect. The presented model is valuable in the search for room temperature operating gas sensors.

## Conclusions

A microchannel with PEDOT:PSS coated walls is an effective VOC filter with sharp selectivity, which can be used to separate ppm-level contaminations from background gases in a microfluidic circuit. This behavior stems from the physicochemical properties of the surface of the functional coating applied to the channel walls, which are amplified by the physics of the microfluidic channel; the filtering action disappears in large cross-section channels. The coated channel transports hydrogen, carbon monoxide, hexane and benzene similar to the uncoated while effectively blocking both diffusion and drift of methanol and ethanol. Separation factor between n-hexane and ethanol is larger than 10^3^. The filtering on ketones is at least 30 times weaker than that observed for ethanol at the same conditions.

The presented model describes the observed selective filtering of the PEDOT:PSS-coated microchannels based on the hydrogen bonding between the contaminating molecules and the coated channel walls: alcohols form hydrogen bonds with both proton acceptor and donor cites while ketones bond only to the donor cites available on the coating surface. The rest of the examined target molecules interact with the coated walls due to the permanent or random dipole-dipole forces which appear to have insignificant consequences at room temperature.

Understanding the mechanism of the gas adsorption to the functional channel wall coating facilitates pinpointing other coatings for the fabrication of different microfluidic filters. Such microchannels coupled to appropriate sensors can be considered for numerous applications in selective chemical sensing and biomarker detection. At its present configuration the introduced device can be utilized for detecting low levels of carbon monoxide or benzene in highly alcohol-contaminated background atmosphere. The presented model is anticipated to be utilized for designing novel resistive chemical sensors based on organic conductors.

## Methods

The microchannels are fabricated by CO_2_ laser engraving on PMMA substrates. Each channel has an inlet at one end and a microcavity which houses a tin oxide-based resistive chemical sensor chip at the opposite end. The geometry and the dimensions of the fabricated channels are given in Fig. 1a. The performance of the PEDOT:PSS-coated microchannel strongly depends on its length and cross-sectional height, which are determined based on the required specifications. The channel width, however, is determined according to the width of the sensing element utilized. The presented geometry was designed for the current context of use (filtering CO from alcohols and ketones) based on our multiple experiments carried out on the channels of different geometries and mathematical work on the diffusion-adsorption of a trace gas in capillaries^52^. The channel design task was omitted from this report for the sake of brevity, but some of the related data can be found as Supplementary Fig. S4a-h.

**Figure 6:**
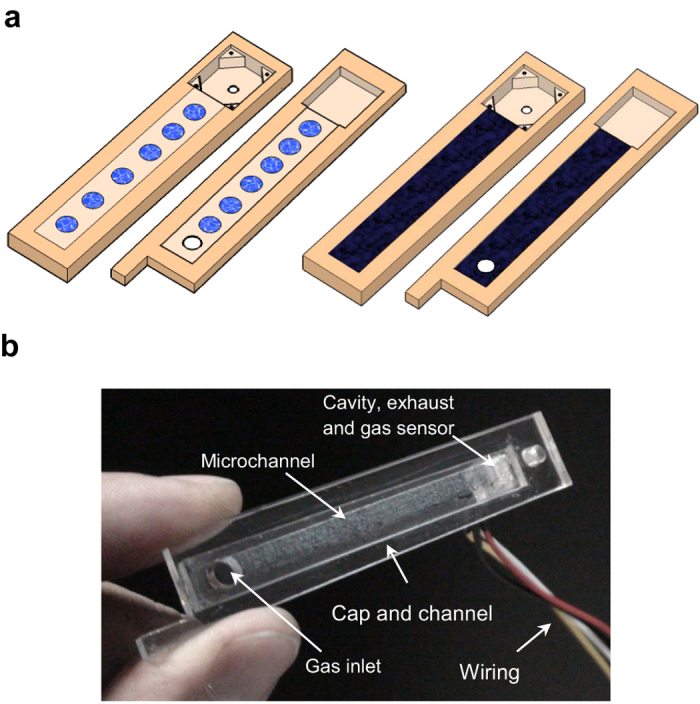
The steps of the fabrication process. (**a**) The schematics of the laser engraved PMMA microchannel demonstrating PEDOTT:PSS suspension droplets casted on the channel walls before and after heat-assisted spreading and drying of the suspension. (**b**) The photograph of a ready-to-use PEDOT:PSS-coated microchannel.

The methodology used for the integration of the sensor chip and the microchannel on the PMMA chip is as described in reference^5^. PEDOT:PSS dispersed in water with 1.3 w% concentration (Sigma-Aldrich, product no. 483095,) is used as-purchased. The suspension is applied by drop casting the suspension to the channel walls heated to 60 °C on a hot plate. Substrate heating allows even surface spreading of the suspension. The substrate and its cap are allowed to dry at room temperature in air overnight. The resulted polymer coating is in the 3–4 μm thickness range (see the insets given in Fig. 1a). The coating process is schematically presented in Fig. 6a, and the photograph of a ready-to-use PEDOT:PSS-coated channel is given in Fig. 6b.

**Figure 7:**
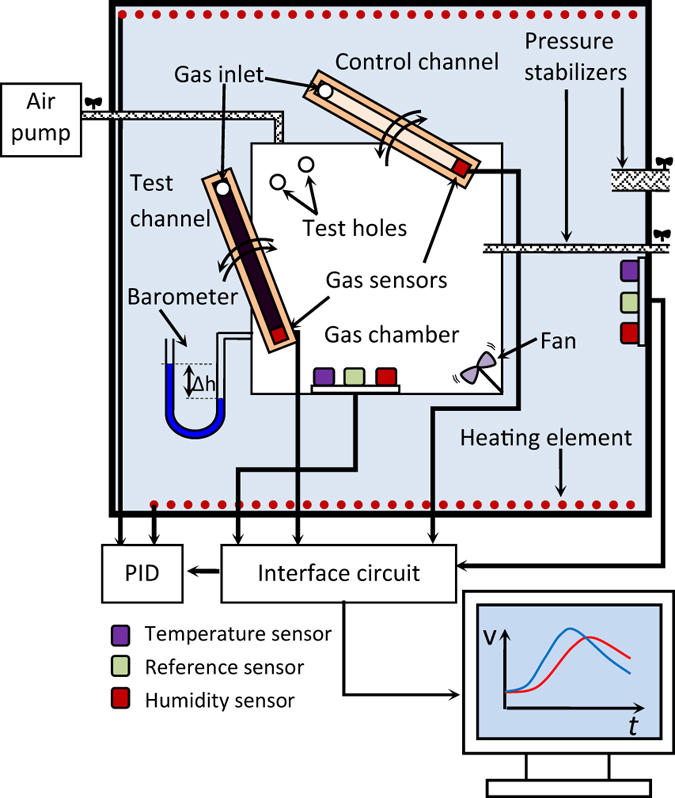
The block diagram of the experimental layout utilized for response recordings.

The layout of the measurement system is schematically given in Fig. 7. The microchannel can rotate around a hinged point on the cap of a chamber with predetermined contaminated atmosphere, so that its inlet gets exposed to the contaminated atmosphere of the chamber through a hole on the cap. The reverse rotation of the channel re-exposes its inlet to the clean air. The background atmosphere in the chamber and the microchannels is clean air at 25.0 °C and 22 + /-1% relative humidity. The air in the chamber is contaminated with the target gas at the predetermined level. A miniature air pump with adjustable power pumps the target gas-contaminated air from a larger reservoir to the test chamber and establishes a positive pressure therein. The positive pressure in the chamber causes a gas flow in the microchannel; the relationship between the positive pressure in the chamber and the established flow rate within the microchannel is determined prior to the main experiments. Chamber pressure is monitored with a homemade U-shaped manometer able to display the pressure differences in the 0.1–3.0 Torr range. The block diagram of the system utilized for recording the sensor generated transient responses is also presented in Fig. 7.

## Additional Information

**How to cite this article**: Hossein-Babaei, F. and Zare, A. H. The selective flow of volatile organic compounds in conductive polymer-coated microchannels. *Sci. Rep.* **7**, 42299; doi: 10.1038/srep42299 (2017).

**Publisher's note:** Springer Nature remains neutral with regard to jurisdictional claims in published maps and institutional affiliations.

## Supplementary Material

Supplementary Information

## Figures and Tables

**Table 1:** The estimated filtering power of the PEDOTT:PSS-coated microchannel shown in Fig. 1a with respect to the stated contaminants along with their molecular dipoles (p)^59^ and diffusion coefficients in air at room temperature (D) 60.

Contaminant	Formula	D [10^−4^ m^2^/s]	p [D]	F
Hydrogen	H_2_	0.6100	0	1
Carbon Monoxide	CO	0. 1920	0.11	1
Methanol	CH_3_OH	0.1528	1.70	>800
Ethanol	C_2_H_5_OH	0.1188	1.69	~9000
Iso-propanol	C_3_H_7_OH	0.1019	1.58	>800
Iso-butanol	C_4_H_9_OH	0.0885	1.64	>800
Acetone	C_3_H_6_O	0.1049	2.88	30
2-Pentanone	C_5_H_10_O	0.0793	2.70	4
Hexane	C_6_H_14_	0.0732	0	1
Benzene	C_6_H_6_	0.0932	0	1
