# Supplementary material for: The selective flow of volatile organic compounds in conductive polymer-coated microchannels
Source: Sci Rep. 2017 Feb 13;7:42299. doi: 10.1038/srep42299 (PMC5304218; doi:10.1038/srep42299)
Supplement: Supplementary Information [file srep42299-s1.pdf]

Supplementary Information

The selective flow of volatile organic compounds in conductive polymer-coated microchannels

Faramarz Hossein-Babaei[1,*] and Ali Hooshyar Zare[1]

[1]Electronic Materials Laboratory, Electrical Engineering Department, K. N. Toosi University of Technology, Tehran 16317-14191, Iran
fhbabaei@kntu.ac.ir

1. Background

Research on the interaction of gas molecules with the channel walls has been in progress in our laboratory for 12 years[52]. We have been aware of the importance of the wall material, and have previously used channels made of borosilicate glass[6], PMMA[5], and stainless steel for different purposes. The idea of channel coating was introduced ~4 years ago. The primary goal was to fabricate a channel for the selective flow of carbon monoxide. We examined a large number of materials for channel coating, ranging from metal and metal-oxide thin films to carbon related nano-composites. Different coatings demonstrate different operational characteristics, but the findings were not significant; the observed transportation parameter differences for different gases were in the percentage range rather than the order of magnitude. Some of our preliminary results are shown in Fig. S1a-d. We subsequently decided to examine a number of organic electronic coating materials. Oxidized poly[2-methoxy-5- (2-ethylhexyloxy)-p-phenylene vinylene] (MEH:PPV)[S1, S2] resulted in responses similar to our uncoated PMMA channels, and the second in line was PEDOT:PSS. The reason for selecting PEDOT:PSS was the sensitivity of its electrical conductivity to a number of volatile organic compounds at room temperature[55-57]. This is, arguably, the sign of a profound surface adsorption process. Based on the model presented in this manuscript, numerous suitable organic materials can be selected and examined for channel coating; polyaniline, polyacetylene, polypyrrole, polythiophene are examples.

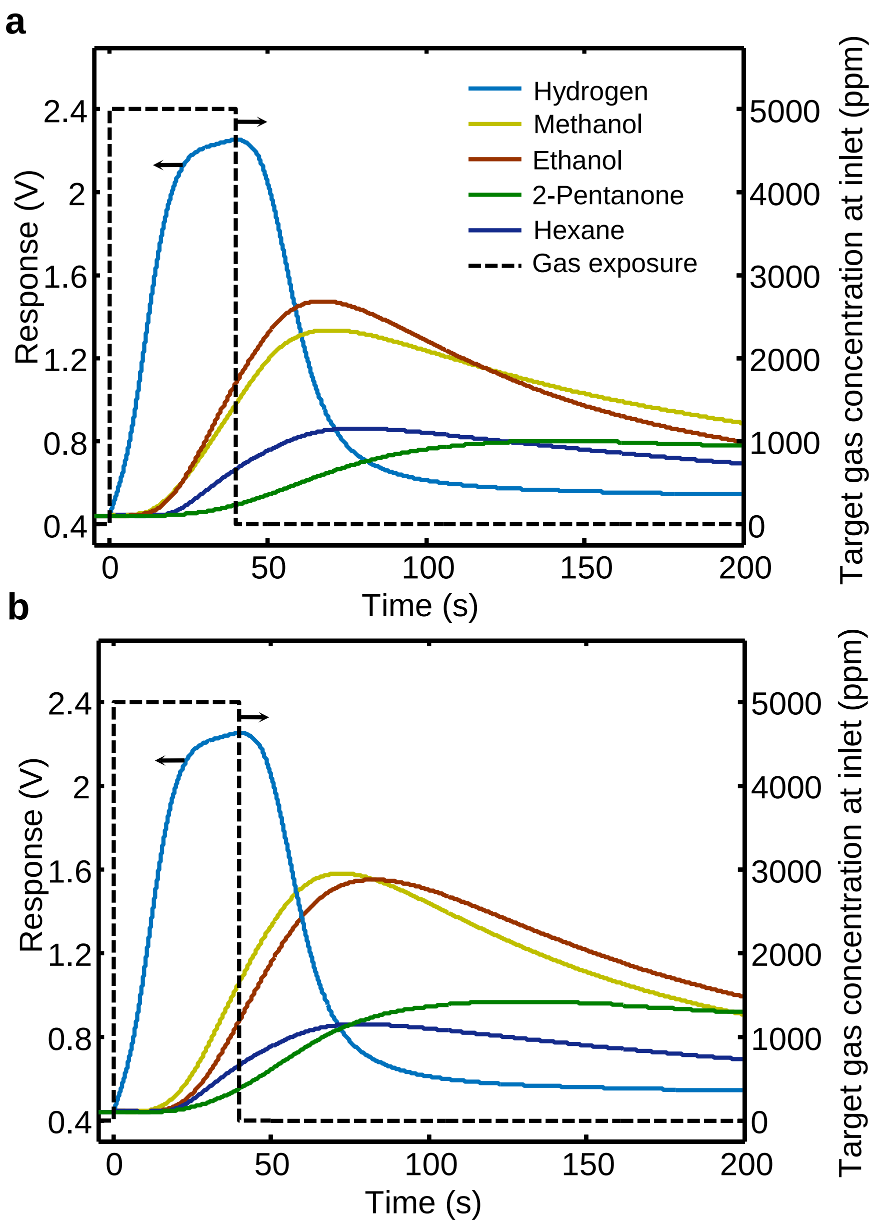
a
b
Hydrogen
Methanol
Ethanol
2-Pentanone
Hexane
Gas exposure
Response (V)
Target gas concentration at inlet (ppm)
Time (s)
0.4
0.8
1.2
1.6
2
2.4
0
1000
2000
3000
4000
5000
0
50
100
150
200

c

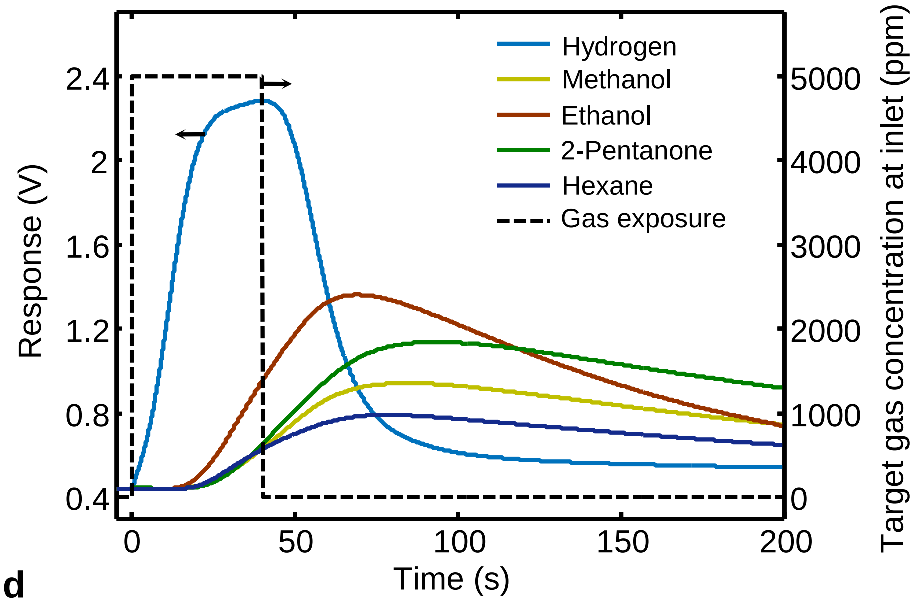

d

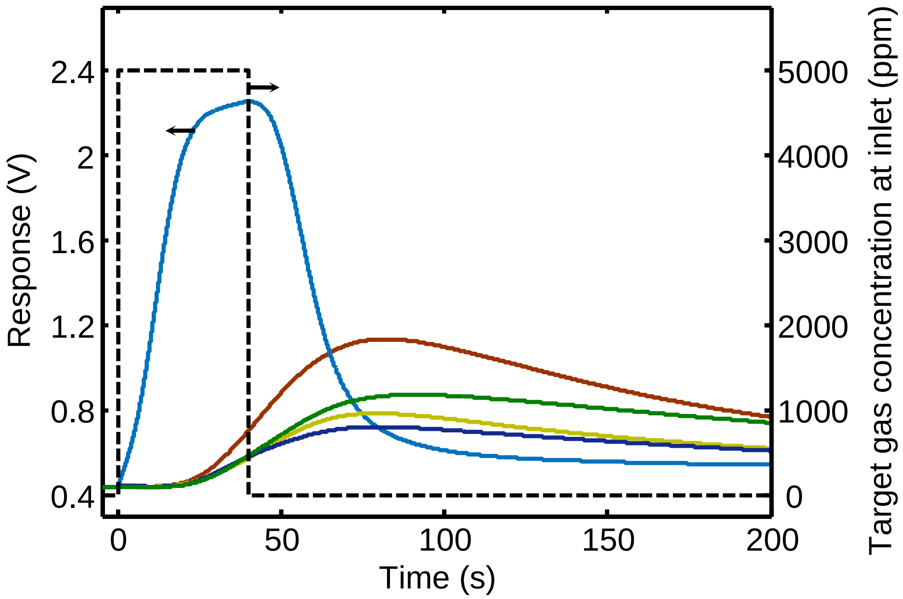

Figure S1. Temporal responses recorded at the channel outlet when the inlet is exposed to air contaminated with the stated contaminants using channels made of PMMA coated with (**a**) Au, (**b**) Ag, and (**c**) oxidized MEH:PPV; the results for the uncoated channel is given in (**d**). The dotted line shows the contaminant variation imposed to the channel inlet. Identical geometrical parameters and experimental conditions are used.

2. Repeatability and Reproducibility of the results

The responses recorded using both the PEDOT:PSS-coated and the control channels are repeatable. The main sources of error in different recordings using a single channel are the temperature and relative humidity variations in the ambient air, which are controlled at 25.0 ± 0.1 ^{o}C and 22 ± 1 %, respectively. The results of 8 recordings carried out at 8 different work sessions utilizing the test and controlled samples are given in Fig. S2 a and b, respectively, showing the similarity of the recorded response profile for each target gas. Reproducibility of the results from one device to another, however, depends on the geometrical accuracy in channel fabrication. The accuracy of the CO_2 laser machining system used in PMMA channel fabrication is ± 5 µm or ± 4 %, whichever is larger, for cross-sectional dimensions and ± 0.5 mm for length. As the coating does not affect hydrogen and hexane transport through the channel, matching of the responses recorded for both hydrogen and hexane in the test and control channels indicates reproducibility of the responses in two different samples; differences in the sensing element calibration cause minor temporal shifts and amplitude alterations, but the gas filtering performance of the device remains intact. Aging of the device does not affect the gas filtering power of the coated channels either: PEDOTT:PSS-coated channel samples kept in clean dry air and operated once a week since 10 months ago operates like new.

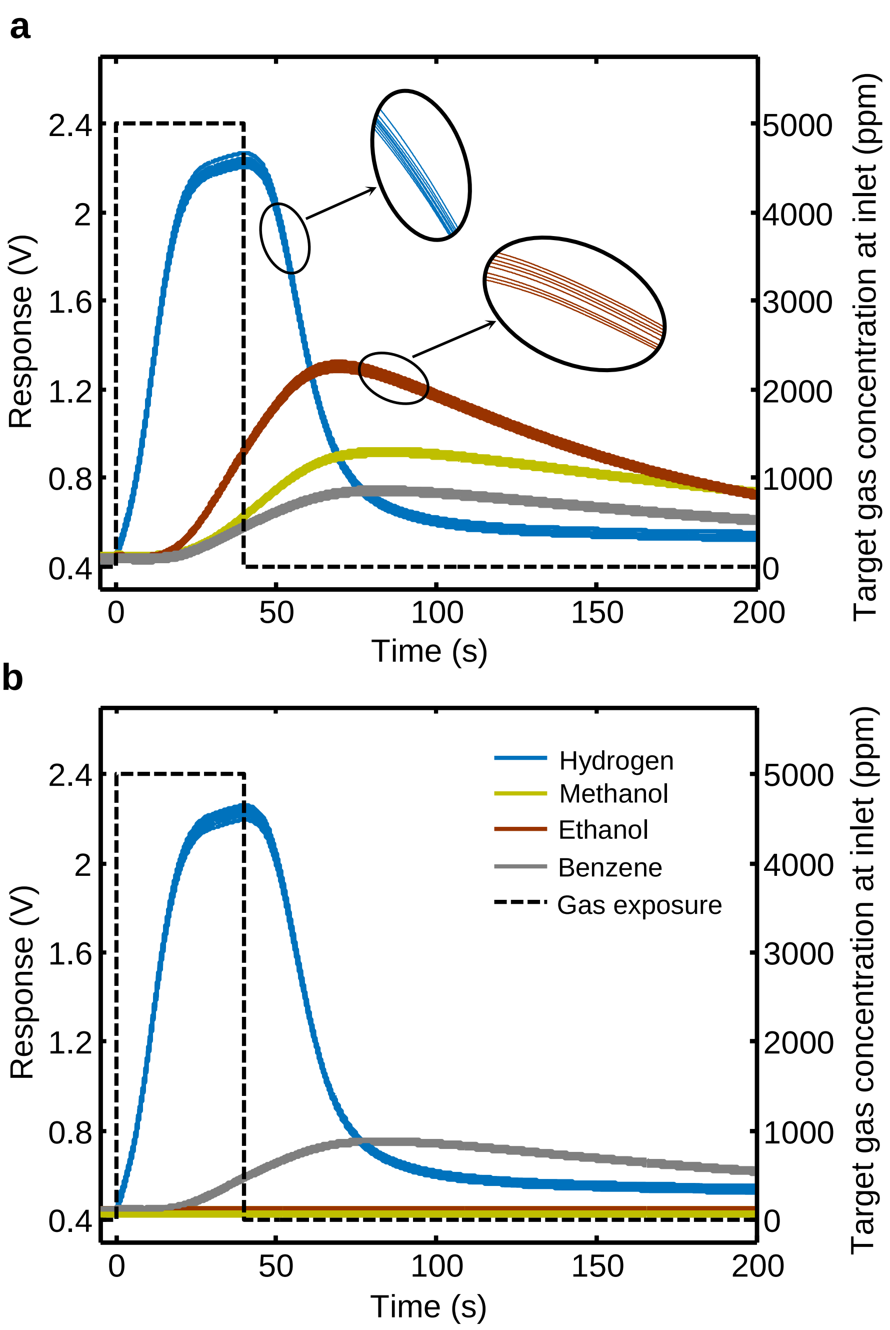

Figure S2. The temporal responses recorded for the stated target gases utilizing the test (a) and control (b) microchannels. The response profiles, obtained by repeating the experiments 8 times in 8 different sessions (see the insets), demonstrate the repeatability of the results.

3. Adsorption Saturation

According to the presented model, the wall surface is expected to gradually saturate with the target gas molecules. This would manifest itself in the recorded channel transportation results as a gradual rise in the target gas response. In the case of acetone, the signs of gradual move towards adsorption saturation has appeared after 100 s (see Fig. 2c), but no sign of saturation was detected for ethanol within 2000 s of inlet exposure (see Fig. 2c). Another experiments was devised, which allowed 4 h of continuous exposure to ethanol contaminated air. The result is presented in Fig. S3, indicating signs of ethanol adsorption saturation after 7000 s.

The response level for ethanol even 2000 s after exposure is immeasurably small. As a result, F_{eth} remained undetermined. A rough estimation of this parameter is afforded by fitting of a mathematical function to the ethanol response profile presented in Fig. S3. The best fitting is achieved using the following function.

$$\boldsymbol{f}(t) = 1.0096 + 0.57 * \boldsymbol{erf}\big(0.00027 * (t - 8800)\big) \qquad (S1)$$

wherein t is the time stated in seconds and ***erf*** stands for the error function. Eq (S1) allows calculation of the response level to ethanol at 235 s and, hence, affords calculation of F_{eth} based on the data given in Fig. 2c. The result is ~9000 (see Table-1).

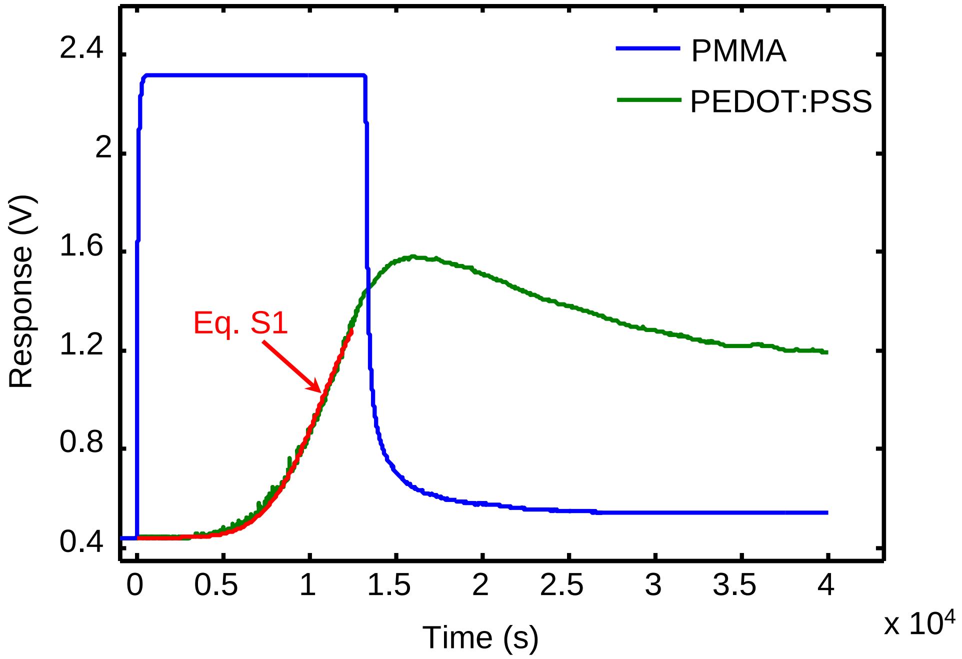

Figure S3. Temporal response profiles obtained by the extended exposure of the test (green) and control (blue) channels inlets to ethanol. The red line is the fitting of (S1) to the ethanol response of the PEDOT:PSS-coated channel.

4. Channel Design

The filtering power of a PEDOT:PSS-coated channel is a function of the length and cross-sectional area of the channel[52]. The design parameters are the channel length and the cross-sectional height, which are determined based on the required specifications. The channel width, however, is fixed according to the width of the utilized sensing element. The presented geometry (Fig. 1a) was designed for the current context of use (filtering CO from alcohols and ketones) based on the results of our multiple experiments carried out on the channels of different geometries; examples are given in Fig. S4. The mathematics of the diffusion-adsorption of trace gases in capillaries[52] provided the background intuition which limited the number of fabricating and experimenting attempts. The criterion for selecting a specific design (Fig. S4g) was its ability to provide discriminable CO and ethanol response profiles at the uncoated state.

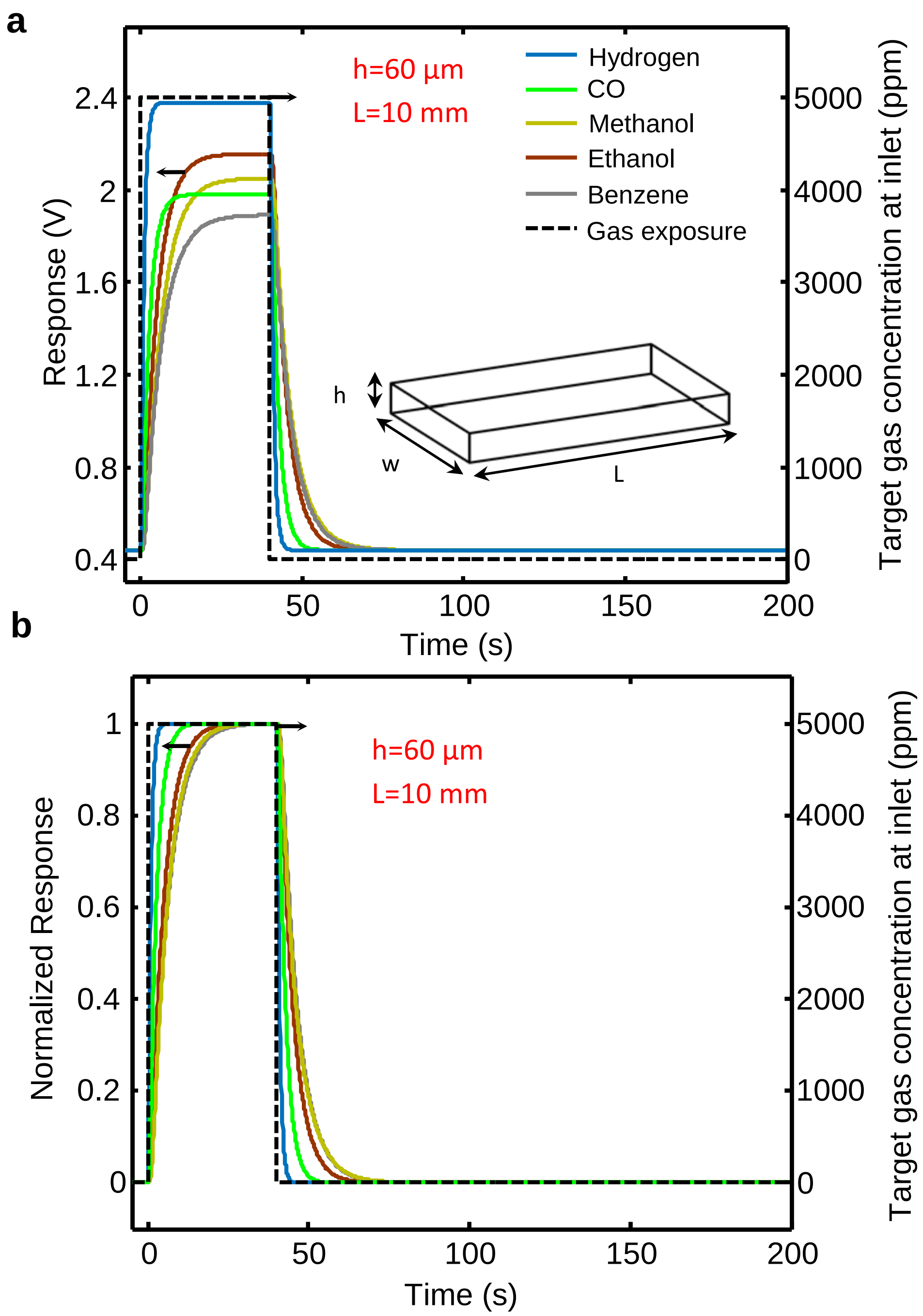
a
h=60 µm
L=10 mm
Hydrogen
CO
Methanol
Ethanol
Benzene
Gas exposure
h
w
L
Response (V)
Target gas concentration at inlet (ppm)
Time (s)
b
h=60 µm
L=10 mm
Normalized Response
Target gas concentration at inlet (ppm)
Time (s)

c

d

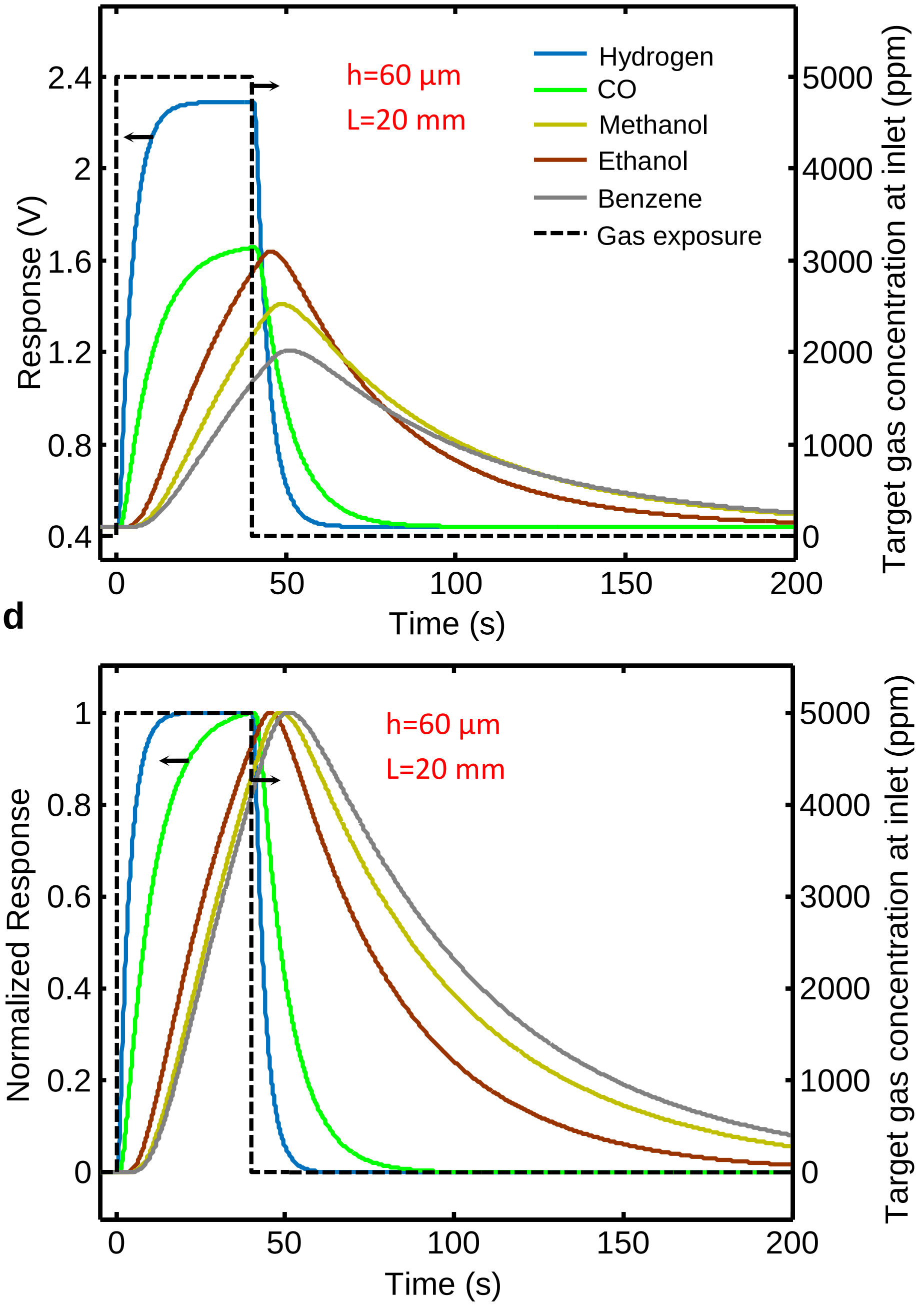

2.4
2
1.6
1.2
0.8
0.4
Response (V)
5000
4000
3000
2000
1000
0
Target gas concentration at inlet (ppm)
h=60 µm
L=20 mm
Hydrogen
CO
Methanol
Ethanol
Benzene
Gas exposure
0
50
100
150
200
Time (s)
1
0.8
0.6
0.4
0.2
0
Normalized Response
5000
4000
3000
2000
1000
0
Target gas concentration at inlet (ppm)
h=60 µm
L=20 mm
0
50
100
150
200
Time (s)

e

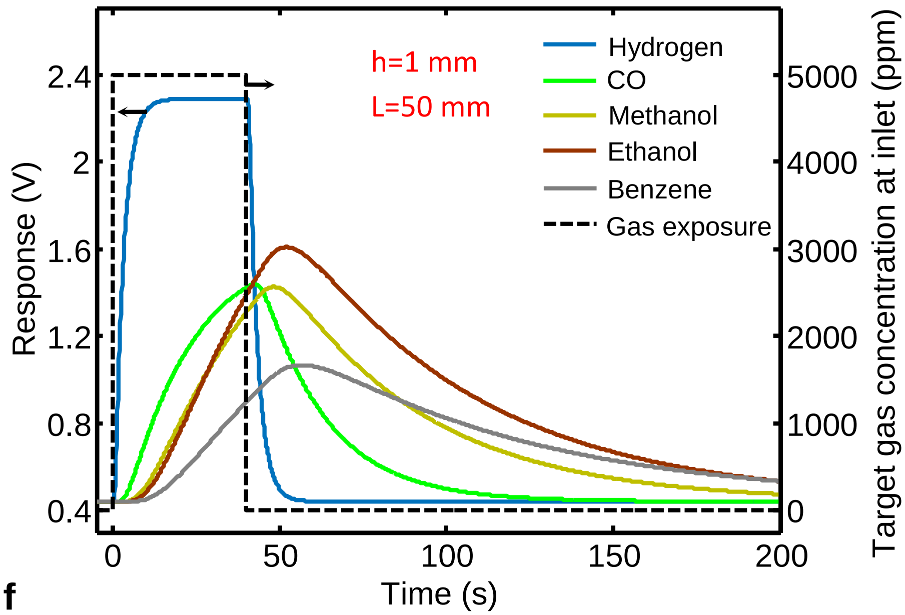
h=1 mm
L=50 mm
Hydrogen
CO
Methanol
Ethanol
Benzene
Gas exposure
Response (V)
Target gas concentration at inlet (ppm)
Time (s)

f

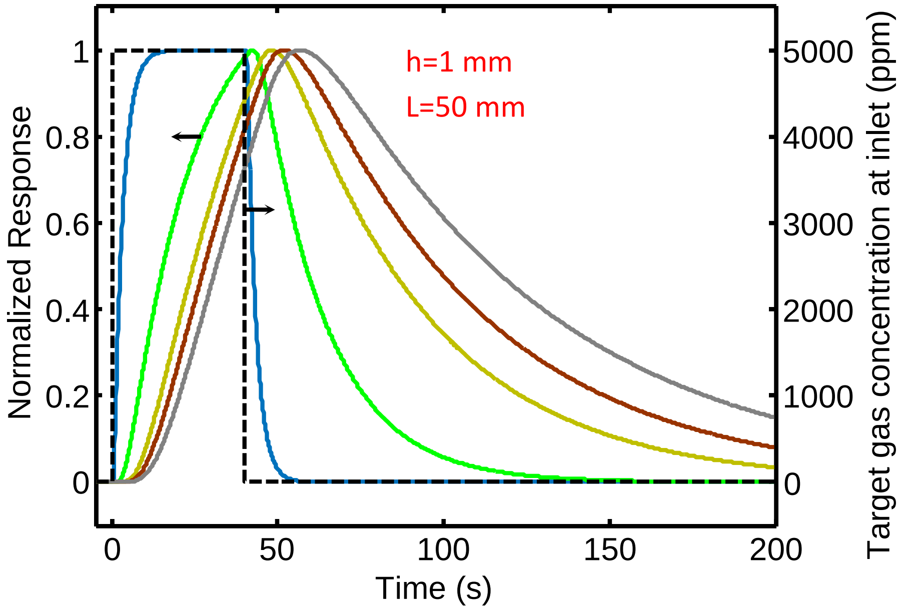
h=1 mm
L=50 mm
Normalized Response
Target gas concentration at inlet (ppm)
Time (s)

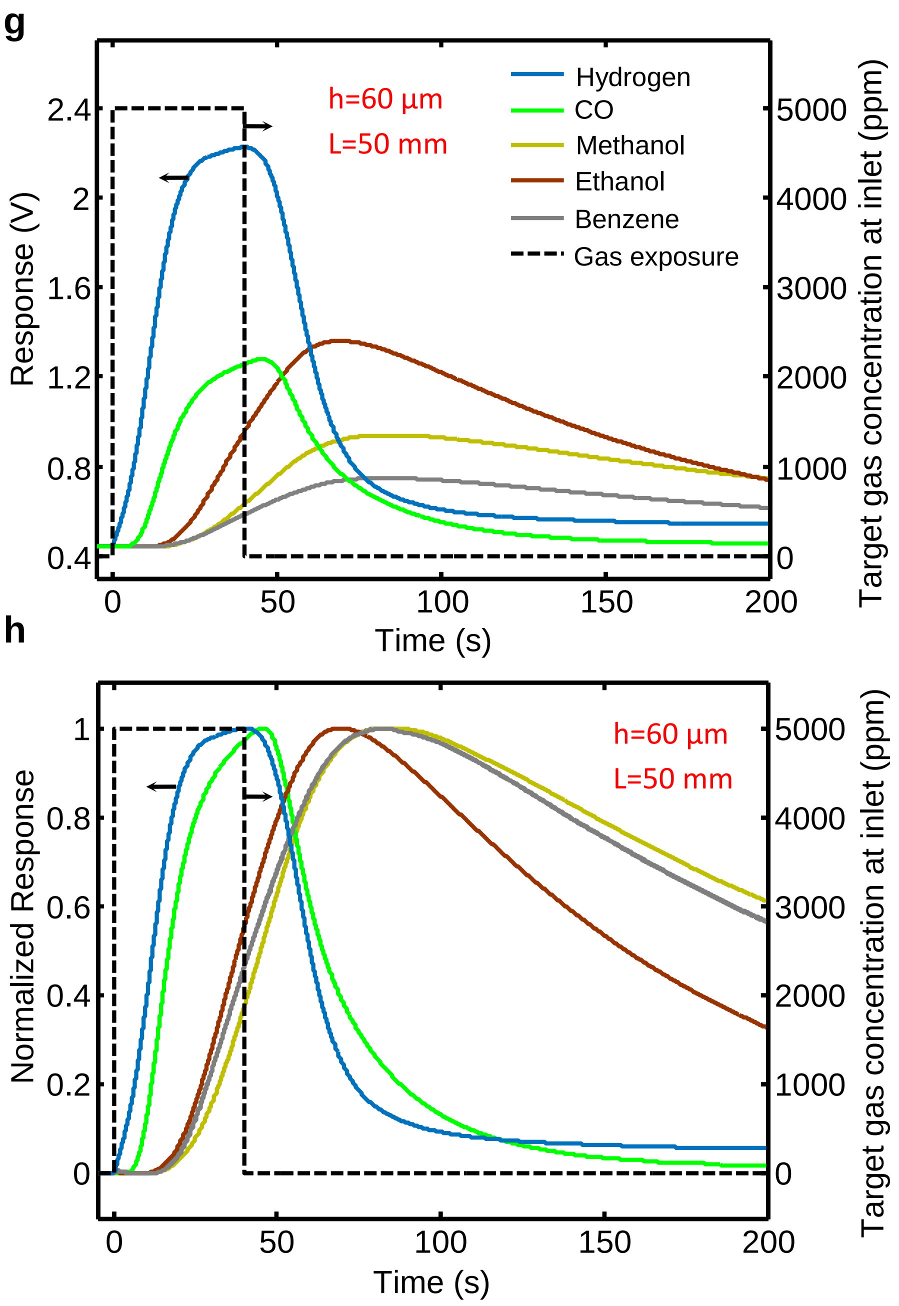

Figure S4. Response profiles recorded for 5 different gases utilizing channels of different dimensions before (a, c, e, g) and after (b, d, f, h) amplitude normalization.

References

S1. Hossein-Babaei, F., Shabani, P. & Azadinia, M. Air-stable electrical conduction in oxidized poly [2-methoxy-5-(2-ethylhexyloxy)-p-phenylene vinylene] thin films. *Appl. Phys. Lett.* **103**, 223303 (2013).

S2. Hossein-Babaei, F. & Shabani, P. A gold/organic semiconductor diode for ppm-level humidity sensing. *Sens. Actuators, B* **205**, 143-150 (2014).
